# Endovascular Baroreflex Amplification for Resistant Hypertension

**DOI:** 10.1007/s11906-018-0840-8

**Published:** 2018-05-09

**Authors:** Monique E. A. M. van Kleef, Mark C. Bates, Wilko Spiering

**Affiliations:** 1 Department of Vascular Medicine, University Medical Center Utrecht, Utrecht University, P.O. Box 85500, 3508 GA Utrecht, The Netherlands; 2 CAMC Research Institute and West Virginia University, Charleston, WV USA

**Keywords:** Resistant hypertension, Sympathetic activity, Baroreceptor, Device-based antihypertensive therapy, Baroreflex activation therapy, Endovascular baroreflex amplification

## Abstract

**Purpose of review:**

Most hypertension devices have been designed to interrupt or modify the sympathetic nervous system, which seems to be unbalanced in hypertension. Carotid baroreceptors play a pivotal role in maintaining adrenergic balance via a direct feedback interface and would be an exceptional target for intervention. The purpose of this review is to define the role of the baroreceptor in hypertension, to examine device-based therapies targeting the baroreflex and to explore future promises of endovascular baroreflex amplification (EBA).

**Recent findings:**

In the last two decades, two therapeutic strategies targeting the carotid baroreceptor have evolved: baroreflex activation therapy (BAT) and EBA. Both therapies enhance baroreceptor activity, either directly by electrical stimulation or indirectly by changing the geometric shape of the carotid sinus and increasing pulsatile wall strain.

**Summary:**

By showing a significant, sympathetic inhibition-mediated effect on blood pressure, BAT has laid the foundation for baroreflex-targeting therapies for resistant hypertension. EBA is a less invasive therapy with promising first-in-man study results. Ongoing randomized sham-controlled trials are needed to better understand efficacy, durability, and long-term safety and define phenotypes that may most benefit from this treatment.

**Electronic supplementary material:**

The online version of this article (10.1007/s11906-018-0840-8) contains supplementary material, which is available to authorized users.

## Introduction

Approximately 10–15% of all treated hypertensive patients have resistant hypertension. Resistant hypertension is defined as blood pressure (BP) that remains above target despite adequate treatment with three or more antihypertensive medications, including a diuretic, or treatment with four or more antihypertensive medications irrespective of BP status [1•]. Patients with resistant hypertension are at increased risk of target organ damage and cardiovascular events [2]. Although contemporary hypertension best medical therapy is often effective, some patients do not respond or cannot tolerate the prescribed antihypertensive medication regimen. This unmet need led to a growing interest in device-based therapies as an alternative or adjuvant to drugs. An exceptional target for these device-based therapies is the arterial baroreflex, which plays a central role in the development as well as propagation of the hypertensive continuum [3•].

Stimulation of the carotid baroreceptor leads to increased baroreceptor firing and reduced sympathetic outflow, resulting in a decrease in BP. Baroreflex activation therapy (BAT), in which a surgically implanted electrical stimulator activates the baroreceptor on the outer carotid sinus wall, is one of the device-based therapies that exploits this feedback loop. Although some efficacy endpoints were positive in a randomized, sham-controlled trial [4••], there are considerable disadvantages: the procedure is invasive, costly, has numerous adverse effects [5], and the battery needs replacement every 3 to 5 years. Endovascular baroreflex amplification (EBA), in which an endovascular device implanted inside the carotid sinus modulates baroreceptor activity, is a less invasive therapy targeting the baroreflex. The first safety and efficacy results of EBA are promising [6••]. This review will focus on baroreceptor stimulation to lower BP in patients with resistant hypertension. First, we will outline the problem of resistant hypertension and provide a clinical framework for the application of device-based therapies targeting the carotid baroreceptor. Thereafter, we will discuss the role of the baroreceptor in hypertension and elaborate on the two therapies targeting the baroreflex: BAT and EBA.

## Resistant Hypertension and Sympathetic Nervous System

The term resistant hypertension was initially introduced to define a group of high risk patients in which screening for secondary causes of hypertension as well as intensified treatment should be considered [1•]. Resistant hypertension is defined as BP that remains above target despite treatment with three or more antihypertensive medications of different classes, including a diuretic, or treatment with four or more antihypertensive medications of different classes irrespective of BP status [7]. Prevalence of resistant hypertension ranges from 8.4 to 17.4% in patients medically treated for hypertension [8–17] and from 8.9 to 12.8% [14–17] in all hypertensive patients. The hypertension device trials have led researchers to better define “resistant hypertension.” Yet, this disambiguation process has been challenging. For example, there are patients with so-called pseudo-resistant hypertension: patients who appear to be resistant to therapy due to white coat effect or non-adherence to medication. The prevalence of white coat effect (ambulatory BP measurements being normal) among patients with resistant hypertension is estimated at 35.7% [18]. Also, the prevalence of non-adherence is surprisingly high in this population. Recent studies using therapeutic drug monitoring (in urine or plasma) to assess non-adherence to antihypertensive medication report percentages of 53 to 68% [19, 20].

It is important to identify pseudo-resistance early in the clinical decision-making process. Cardiovascular risk in patients with true resistant hypertension is at least two times as high compared to those with controlled ambulatory BP [2]. Therefore, classification identifies patients at higher risk and justifies treatment intensification. Moreover, this classification is crucial to distinguish the patients that may benefit from expensive and potentially harmful device-based treatments from those in which alternative strategies, i.e., improving medication adherence, should be initiated. To date, the ideal study estimating the prevalence of true resistant hypertension has not yet been conducted. However, considering the aforementioned numbers of pseudo-resistance, prevalence of true resistant hypertension will not be much higher than 5–10%, which still can be extrapolated to tens or hundreds of million patients worldwide.

Resistant hypertension is characterized by increased sympathetic drive [3•]. Studies quantifying sympathetic activity by measuring muscle sympathetic nerve activity (SNA) at the peroneal nerve have shown an increase in SNA in pre-hypertensive stages [21]. SNA progressively increases from mild to severe hypertension [22] and is even more pronounced in patients with resistant hypertension [23•]. Increased SNA is associated with target organ damage and an increased risk of cardiovascular events [3•]. Therefore, the sympathetic nervous system has become one of the most important targets for device-based therapies in the last decade.

## Baroreflex Physiology

As the baroreceptors are important modulators of SNA, they are an exceptional target for antihypertensive therapy. Arterial baroreceptors are mechanosensitive nerve fibers located in the carotid sinus, close to the bifurcation, and in the aortic arch. These nerve fibers are located in a specialized area in the lateral wall of the carotid sinus, opposite the carotid body (another neural structure involved in BP control). The carotid sinus contains more collagen and less smooth muscle cells than the innominate, making it more compliant and contributing to stretch-induced activation of the baroreceptors [24]. Baroreceptors react on stretch rather than pressure. This has been demonstrated in an experimental model of a carotid sinus imbedded in a non-distensible plaster in which baroreceptor firing remained unaffected by increases in arterial pressure [25]. Baroreceptor nerve fibers contain ion channels that are sensitive to mechanical deformation [26•]. Increase in wall strain causes influx of sodium and calcium ions through these channels, depolarizing the nerve terminal and generating action potentials that travel along the afferent nerve [27]. The frequency of firing depends on the magnitude of deformation and the properties of the ion channels and ion pumps located at the nerve terminal. Via the glossopharyngeal and vagus nerves, these signals travel to the nucleus tractus solitarius (NTS) in the caudal medulla. The NTS is the main regulatory center of the BP that integrates multiple information from different organ systems and the cerebral cortex. This central role of the NTS in BP regulation has been demonstrated in rat experiments. Blockage of neurotransmission in the NTS strongly attenuated the baroreflex [28] and specific lesions in the NTS eliminated baroreceptor responses completely [29]. In the NTS, two signaling pathways arise: the parasympathetic and sympathetic pathway. Signals from the parasympathetic pathway travel via the nucleus ambiguous to the heart, where parasympathetic drive is increased. The signals from the sympathetic pathway travel via the caudal ventrolateral medulla, where the signal is conversed into an inhibitory signal, to the rostral ventrolateral medulla (RVLM) decreasing sympathetic output [30]. The RVLM is critical for maintenance of vascular tone and is the primary output nucleus for muscle SNA.

In hypertensive patients, the baroreceptor undergoes complex changes to compensate chronic high BP status. Baroreceptors in hypertensive patients fire at higher pressures compared to normotensives in order to prevent saturation of the reflex and avoid extreme vasoconstriction and tachycardia [3•]. Although the baroreflex control of heart rate (parasympathetic pathway) is impaired [22], the baroreflex control of BP (sympathetic pathway) is unaffected in hypertension [31]. Therefore, the baroreceptors retain their ability to respond to transient changes in BP. This means that there is a shift in baroreflex activation to a higher set point, in other words, the baroreceptors are reset. Although, the specific mechanisms underlying resetting of the carotid baroreflex are yet unknown, it is likely to be multifactorial process including peripheral as well as central mechanisms [32, 33].

Considering the aforementioned mechanisms, modulating the baroreflex to decrease sympathetic activity seems a promising strategy to lower BP. Two device-based therapies that rely on this mechanism are BAT and EBA. BAT increases baroreceptor firing rate by directly delivering electric pulses to the baroreceptor nerve endings on the outer wall of the carotid sinus. EBA relies on mechanically changing the geometric shape of the carotid sinus during cardiac systole, which increases pulsatile strain. Pulsatility is crucial since static increases in BP reset the baroreceptor immediately, where pulsatile strain attenuates baroreceptor resetting [34]. This explains the difference in BP response to conventional carotid stenting, where low BP returns to normal within a few days, and implantation of the dynamic MobiusHD device, where decrease in BP sustains.

## Lessons Learned from Baroreflex Amplification Therapy

Implantation of an electrical stimulator to activate the baroreceptor was already performed in humans in the 1950s [35]. Application of this technique in patients with severe hypertension [36, 37] or angina pectoris [38] showed a significant decrease in BP and relief of angina. Despite these promising results, the interest in electrical baroreceptor stimulation as a treatment strategy for hypertension declined due to the introduction of more effective and better tolerated antihypertensive drugs, and the technical problems associated with the first electrical stimulators. It is only since the last decade that baroreceptor stimulation regained interest for treatment of patients with resistant hypertension.

The Rheos system, an implantable electrical stimulator, has been developed by CVRx and was first investigated in 11 patients undergoing elective carotid endarterectomy [39]. Unilateral electric stimulation of the carotid sinus wall during surgery decreased systolic BP from 144 ± 9 to 131 ± 9 mmHg. The first permanent implantations in humans were performed in a European multicenter feasibility study (the DEBuT-HT study) that included patients with resistant hypertension (office BP ≥ 160 mmHg systolic or ≥ 90 mmHg diastolic despite treatment with at least three antihypertensive medications, including a diuretic) between 2004 and 2007 [40•]. First-generation Rheos stimulation electrodes were placed bilaterally on the outer wall of the carotid sinus and connected to an impulse generator positioned inferior to the clavicle. BP decreased by 21/12 ± 4/2 mmHg at 3 months (*n* = 37) and 33/22 ± 6/4 mmHg after 2 years (*n* = 17). The subsequent randomized double-blind Rheos Pivotal trial could not prove significant acute efficacy (proportion of patients that achieve at least 10 mmHg decrease in systolic BP at 6 months) and procedural safety (serious procedure- or system-related adverse event-free rate within 30 days of implantation) [4••]. However, the study did show a significant difference in the proportion of patients that reached systolic BP ≤ 140 mmHg: 42% in the active treatment group and 23% in the placebo group. Moreover, systolic BP decreased by 26 ± 30 mmHg in the active treatment group versus 17 ± 29 mmHg in the placebo group, at 6 months compared to pre-implantation. Additionally, a 40% reduction of hypertensive events in the active treatment group was observed. Yet, the substantial rate of general surgical complications and nerve injury causing transient or residual symptoms as localized numbness, dysphagia, or dysphonia led to the development of a second-generation device: the Barostim neo. The electrodes of the Barostim neo are substantially smaller in size, and it requires unilateral implantation only.

The Barostim neo has only been investigated in non-randomized studies so far. The first studies showed a significant systolic office BP decrease of 26 ± 4 mmHg (*n* = 30), comparable with the first-generation results [41•], and systolic 24-h ambulatory BP decrease from 148 ± 17 to 140 ± 23 mmHg (*n* = 44) [42] at 6 months. Forty-three percent reached systolic office BP ≤ 140 mmHg [41•]. More importantly, considerably less system- or procedure-related events occurred compared to the first-generation device [41•, 42]. Proof of mechanism studies with first- and second-generation devices have confirmed that depressor response to BAT was mediated through sympathetic inhibition [5, 43••] and have shown that physiologic regulation of the baroreflex remained unaffected [43••]. The Barostim neo is currently being investigated in a randomized double-blind clinical trial: the Nordic BAT study [44].

The aforementioned clinical trials on BAT provide the evidence that baroreflex activation is an effective strategy to lower BP in patients with resistant hypertension. However, due to the current lack of results from randomized double-blind clinical trials, the side effects that occur with increased energy delivery [5], the need for a surgical procedure, the high costs, and the need for battery replacement every 3–5 years, there has been a pursuit for alternative techniques to activate the baroreflex and lower BP.

## Endovascular Baroreflex Amplification

**Fig. 1:**
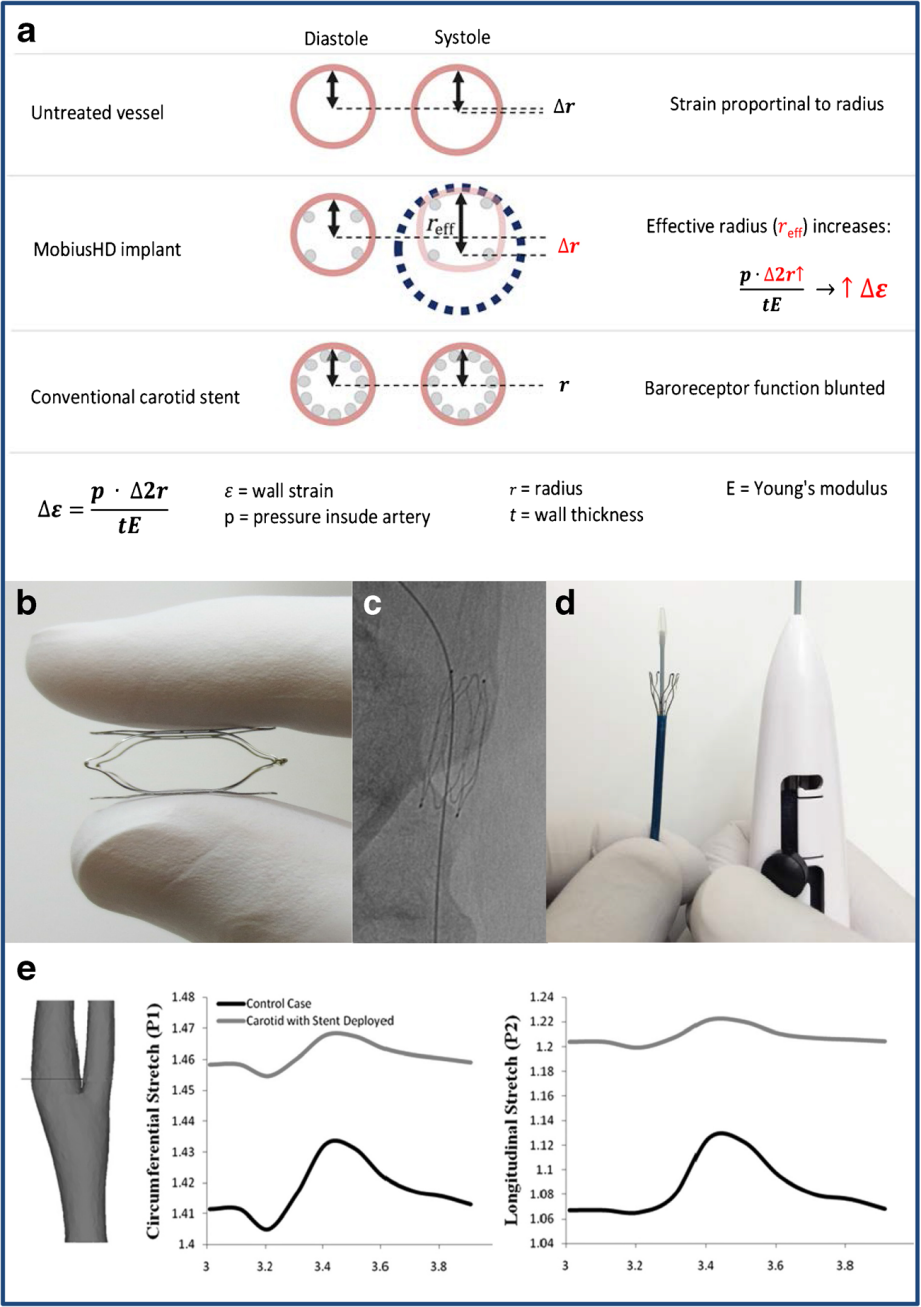
**b**–**d** Images are reproduced by permission of Vascular Dynamics, Inc. **a** Cross-sectional view of the carotid sinus in three different situations. In the untreated vessel, the radius increases pulsatile during the systolic phase of the cardiac cycle. The MobiusHD implant changes the geometric shape of the vessel during the systole and, therefore, increases the effective radius (∆r, in red). This results in increased vessel wall strain while preserving pulsatility. The conventional carotid stent drives the carotid sinus into a static circular shape, blunting baroreceptor function. **b** The self-expendable nitinol MobiusHD device (**c**) implanted in the proximal internal carotid artery. **d** The device is delivered by a specially developed delivery catheter, introduced over a guidewire via the femoral artery. **e** *Reproduced from Peter DA*, *Alemu Y*, *Xenos M. Fluid structure interaction with contact surface methodology for evaluation of endovascular carotid implants for drug-resistant hypertension treatment. Journal of Biomedical Engineering. 2012:134;041001–6. DOI: 0.1115/1.4006339.* Computer simulation showing circumferential and longitudinal wall stretch variation in an average carotid sinus after device implantation, plotted for the plane shown on the left

EBA relies on a passive activation of the baroreceptor by intermittingly changing the geometric shape of the carotid sinus which increases pulsatile wall stretch (Fig. 1a). The MobiusHD is a self-expanding nitinol implant that promotes this geometric shape change. The mechanism of action is not intuitive: the outward radial forces of the longitudinal nitinol struts are not active components of the device, rather the carotid bulb components within the windows of the device. In the systolic phase of the cardiac cycle, the arc of the carotid in each window of the device projects a radius that is much larger than the radius of the baseline carotid bulb without geometric change. According to the strain equation, increased wall strain or vessel stretch would be expected with each systolic phase. In the diastolic phase of the cardiac cycle, quadrants within the device windows return to the baseline exaggerated arc. Therefore, the end result of the MobiusHD is increased carotid sinus pulsatile stretch. The implant is available in three sizes: 5.00–7.00 mm, 6.25–9.00 mm, and 8.00–11.75 mm and is placed into the internal carotid artery by a specially developed delivery catheter introduced over a guidewire via the femoral artery (Fig. 1b–d).

## Animal Studies

The pre-clinical evaluation included several animal models to study the effect of MobiusHD implantation on hemodynamics and vessel anatomy. In a canine model, carotid baroreceptor firing rate was measured after implantation of the MobiusHD and compared to implantation of conventional self-expanding carotid stent [6••]. The carotid stent caused a stair step increase of carotid baroreceptor nerve activity that was decoupled with BP. In contrast, the MobiusHD not only increased immediate baroreceptor firing rate to a greater extent than the conventional carotid stent but, more importantly, showed a progressive linear increase in firing as the animal’s BP was increased (Supplementary Fig. 1A). In addition, these experiments showed that MobiusHD implantation was followed by an immediate drop in BP (Supplementary Fig. 1B). In order to optimize design and ensure radial force could be programmed to reduce vessel injury or device migration through the vessel wall, several ovine carotid survival studies were performed to inform design metrics (data unpublished). Since few animals have carotid baroreceptors and an ideal surrogate for the human carotid artery is lacking, information of the effect of MobiusHD implantation on carotid biomechanics and hemodynamics largely comes from computer simulation studies.

## Computer Simulation Studies

The effect of MobiusHD implantation on the carotid wall and vessel hemodynamics was studied by fluid-structure interaction combined with contact surface methodology. This technology allows for a three-way coupled dynamic interaction simulation of the endovascular device, carotid sinus, and fluid flow [45••]. Simulations were performed in two carotid models: the first representing an average carotid artery in terms of geometry and dimensions and the second representing the clinical worst-case scenario of a carotid bifurcation, devoid of a typical sinus, much smaller in dimensions, and having the internal and external carotid arteries (ICA and ECA) aligned almost parallel to each other (Supplementary Fig. 2A). The simulations showed an increase in carotid wall stretch and strain after MobiusHD deployment, affecting hemodynamics only in extreme situations. In the average model device, placement resulted in increased circumferential and longitudinal wall stretch of 2.5 and 7.5%, respectively. Von Mises wall stress in the ICA increased from 198 to 305 kPa (Supplementary Fig. 2B). The peak stress at the level of the bifurcation and the peak flow velocity in ICA and ECA did not change. In the diminutive model, circumferential and longitudinal wall stretch both increased with 6%. Von Mises wall stress in the ICA increased from 60 to 90 kPa. Device deployment caused a reduction of the ECA inflow area, increasing the velocities and peak stress at the level of the bifurcation: peak stress increased from 211 to 622 kPa. Since increased stretch and stress increases baroreceptor firing rate [46•], these elevations are presumed to activate the baroreflex and reduce BP.

This simulation study additionally showed that pulsatility of the average carotid wall, although moderately attenuated, was maintained [45••] (Fig. 1e). The authors reason that the observed attenuated pulsatility is exaggerated as a result of prespecified characteristics of the device material: modeling was performed with stainless steel properties, being less elastic and stiffer than the nitinol the MobiusHD is made of. Therefore, these studies predict that the MobiusHD implantation preserves pulsatility in the carotid vessel.

## Results from the First-in-Man Study

Two parallel proof-of-principle studies investigated the safety and efficacy of EBA in patients with resistant hypertension: controlling and lowering blood pressure with the MobiusHD, first in man, USA and Europe (CALM-FIM_US and CALM-FIM_EUR). The first MobiusHD implantation in human was performed in 2013, Atlanta, USA. The CALM-FIM_EUR study enrolled the last patient in February 2017, and the results of this study were published in September 2017 (6**••**). The CALM_FIM_EUR study included 30 patients in six European centers: five in The Netherlands and one in Germany. Patients were eligible if they were 18–80 years of age; diagnosed with primary resistant hypertension; taking a stable regimen for at least 30 days with maximally tolerated doses of at least three antihypertensive drugs from different classes (one of them being a diuretic); and having a mean systolic office BP of at least 160 mmHg, mean 24-h ambulatory BP of at least 130 mmHg systolic, and at least 80 mmHg diastolic. The main exclusion criteria were hypertension secondary to an identifiable and treatable cause other than sleep apnea; any plaque or ulceration in the carotid artery or aortic arch; inadequate diameter or anatomy of the carotid vessels; history of orthostatic hypotension or syncope; atrial fibrillation; history of myocardial infarction in the past 3 months; history of cerebral vascular accident in the past 12 months; and severe renal, cardiac, or pulmonary disease. MobiusHD implantation was performed by interventionists that had at least performed 100 carotid stent implants before. Patients were treated with dual antiplatelet therapy aspirin, and clopidogrel (or equivalent), administered 3 days before up to 3 months after the procedure. Aspirin was continued indefinitely.

Patients were on average 52 years old, 15 (50%) were female, 8 (27%) had failed renal denervation, mean office BP was 184/109 mmHg (SD 18/14), mean 24-h ambulatory BP 166/100 mmHg (SD 177/14), and mean number of antihypertensive medications 4.4 (SD 1.4). The primary endpoint was the incidence of serious adverse events (SAEs) and unanticipated device effects (UADEs) at 6 months. In the first 6 months, two patients had to be treated because of severe hypotension, two because of worsening of hypertension (one case in which both hypotension and hypertension occurred), and one because of dislodgement of the femoral closure device. After thromboendarterectomy, this patient developed a wound infection, which was treated by wound irrigation and antibiotics. No UADEs occurred. Most common adverse events were dizziness, musculoskeletal pain, hypotension, and groin hematoma. Not unimportantly, two patients in one center had a transient ischemic attack; fortunately, their symptoms resolved.

Mean office BP (average of two readings after 5 min rest, measured seated with an automated oscillometric device) decreased by 24/11 mmHg (95%CI 12–35/4–18) at 3 months and 24/12 mmHg (95%CI 13–34/6–18) at 6 months. Mean 24-h ambulatory BP decreased by 15/8 mmHg (95% CI 7–23/3–13) at 3 months and 21/12 mmHg (14–29/7–16) at 6 months. This decrease was seen on top of reduction in the number of antihypertensive medication by 0.5 (IQR 1.3–0.0). Unfortunately, only self-reported use of medication was assessed.

Although the sample size is small and the results of this proof-of-principal study can be highly affected by different types of bias, including regression to the mean, Hawthorne-effect, placebo effect, and observer bias, the observed decrease in BP is promising. More information on adverse effects in a larger group of patients is needed before we can reasonably state that the treatment is safe. Moreover, we need to wait for the results of the randomized, sham-controlled clinical trials to prove its efficacy.

## Ongoing Clinical Trials

The CALM-START study (controlling and lowering blood pressure with the MobiusHD—studying effects in a randomized trial) is currently enrolling patients with primary resistant hypertension in planned six centers in the Netherlands and four in Germany. In this randomized sham-controlled trial, patients aged 18–70 on a stable regimen of three to four antihypertensive medications and mean 24-h ambulatory systolic BP of 135–170 mmHg are randomized to MobiusHD implantation or sham. The primary endpoint is change in mean 24-h ambulatory systolic BP at 3 months, measured after antihypertensive medication washout. The CALM-2 study (controlling and lowering blood pressure with the MobiusHD) is a second randomized, sham-controlled multicenter trial studying the effect of EBA on BP and is planned to start recruiting patients in spring 2018. In this study, patients with resistant hypertension and mean 24-h ambulatory systolic BP of 145–200 mmHg on a confirmed stable regimen of three to five maximally tolerated antihypertensive medications (containing at least an angiotensin-converting enzyme inhibitor or angiotensin II receptor blocker, a calcium channel blocker, and a diuretic) will be randomized. The primary endpoint is change in mean 24-h ambulatory systolic BP, obtained after observed drug intake at 6 months. These randomized trials will also provide us with more elaborate information on the efficacy and safety profile of EBA.

In addition, we are currently conducting a proof-of-mechanism study to determine the effect of EBA on SNA and baroreceptor sensitivity, in a sub-study of the CALM-DIEM study (controlling and lowering blood pressure with the MobiusHD—defining efficacy markers). In this study, patients with therapy-resistant hypertension who are eligible for MobiusHD implantation undergo microneurography, cardiovascular measurements, and functional magnetic resonance imaging at baseline and 3 months, after washout of antihypertensive medications that influence sympathetic activity. Hopefully, this study will give us insight in the physiological mechanisms by which EBA reduces BP and may help us to identify which patients benefit most from treatment.

## Possible Benefits beyond Blood Pressure

In addition to the effect on BP and the associated decrease of target organ damage, reduced sympathetic activity may have direct beneficial effects on cardiac function, renal function, and insulin sensitivity. From prospective studies in patients with primary hypertension [47] and patients with heart failure [48, 49], we know that sympathetic over-activity is associated with left ventricular hypertrophy, cardiac arrhythmias, and progressive heart failure, independent of BP. Therefore, baroreceptor stimulation may also be beneficial for patients with heart failure. A non-randomized study investigated the effect of BAT in patients with New York Heart Association functional class III heart failure and showed favorable effects on subjective (quality of life) and objective (left ventricular ejection fraction and BNP) heart failure-related outcomes [50]. A beneficial effect of sympathetic inhibition was also observed in one of our study participants, who exhibited a sharp reduction of premature ventricular complexes, 3 months after EBA.

Furthermore, sympathetic inhibition may counteract the negative effects of sympathetic outflow to the kidneys: renin release, sodium reabsorption, and renal vasculature changes [51] (including smooth muscle cell proliferation and vasoconstriction) leading to proteinuria and glomerulosclerosis [52]. Sympathetic inhibition may slow down progression of renal disease or improve renal function. In a non-randomized, single-center study, 23 patients with chronic kidney disease stage 3 or higher treated with the Barostim neo showed reduced proteinuria compared to patients who did not receive BAT [53]. However, no difference in estimated glomerular filtration rate was observed.

Finally, EBA may play a role in regulating glucose metabolism. From earlier studies, we know that impaired glucose metabolism and hypertension often coexist. Most likely, hyperinsulinemia is preceded by increased sympathetic activity. The evidence comes from a prospective cohort study in Japan, which followed 662 normotensive and 188 borderline hypertensive age- and body mass index-matched patients for 10 years [54]. One of the explanations for the occurrence of hyperinsulinemia is that sympathetic activation leads to vasoconstriction, which lowers skeletal muscle blood flow and reduces glucose delivery to skeletal muscles [55] [56]. The effect of baroreflex stimulation on insulin sensitivity was investigated in a randomized, double-blind, cross-over trial in BP responders treated with BAT. Acute change of chronic baroreceptor stimulation did not significantly change muscular glucose delivery and insulin sensitivity [57]. Whether EBA improves cardiac function, renal function, and insulin sensitivity remains to be determined.

## Conclusions

From a pathophysiological point of view, the carotid baroreceptor is an exceptional target to treat hypertension. This has been confirmed in the clinical trials evaluating BAT, showing a significant, sympathetic inhibition-mediated decrease in BP. Therefore, BAT has laid the foundation for baroreflex-targeting devices as an additive treatment for true resistant hypertension. As amplification of the baroreflex by a passive endovascular implant is less invasive, probably less costly, and does not need battery replacement every 3–5 years, EBA may be a good alternative. Although the results of the first-in-man study are promising, efficacy, durability, and safety results from randomized sham-controlled clinical trials are needed before it can be implemented as a standard medical therapy. Furthermore, future research should address which patients benefit most from EBA and should examine the additional effects of sympathetic inhibition beyond lowering BP.

## Electronic Supplementary Material

Supplementary Figure 1 Reproduced from Spiering W, Williams B, van der Heyden J. Endovascular baroreflex amplification for resistant hypertension: a safety and proof-of-principle clinical study. Lancet. 2017;390(10113):2655–61(Fig. S1-S2). DOI: https://doi.org/10.1016/S0140-6736(17)32337-1. Effect of MobiusHD implantation in canine model. (A) Firing rate of the canine baroreceptor after implantation of the MobiusHD device (red) compared to a conventional carotid artery stent (blue) at different blood pressure levels. Maximal nerve activity as well as the baroreceptor response to increases in blood pressure is higher after MobiusHD implantation. (B) After deployment of the first MobiusHD device (first arrow), systolic and diastolic BP drop immediately. Deployment of a second MobiusHD device in the contralateral carotid sinus (second arrow) does not lower BP any further. Heart rate remains unchanged. BP = blood pressure. CAS = carotid artery stent. (JPEG 619 kb)

Supplementary Figure 2 Reproduced from Peter DA, Alemu Y, Xenos M. Fluid structure interaction with contact surface methodology for evaluation of endovascular carotid implants for drug-resistant hypertension treatment. Journal of Biomedical Engineering. 2012:134;041001–2/5. DOI: 0.1115/1.4006339. (A) The two models of the carotid bifurcation used to perform simulations: the left representing an average carotid artery, the right representing the clinical worst-case scenario devoid of a typical sinus, smaller in dimensions and internal and external carotid arteries aligned almost in parallel position. (B) Wall stress distribution (mapped in units of Pa) with the MobiusHD implanted in the carotid sinus of the two models, showing the regions of high wall stress. (JPEG 612 kb)
