# Supplementary figures and images for: Endovascular Baroreflex Amplification for Resistant Hypertension
Source: Curr Hypertens Rep. 2018 May 9;20(5):46. doi: 10.1007/s11906-018-0840-8 (PMC5942348; doi:10.1007/s11906-018-0840-8)

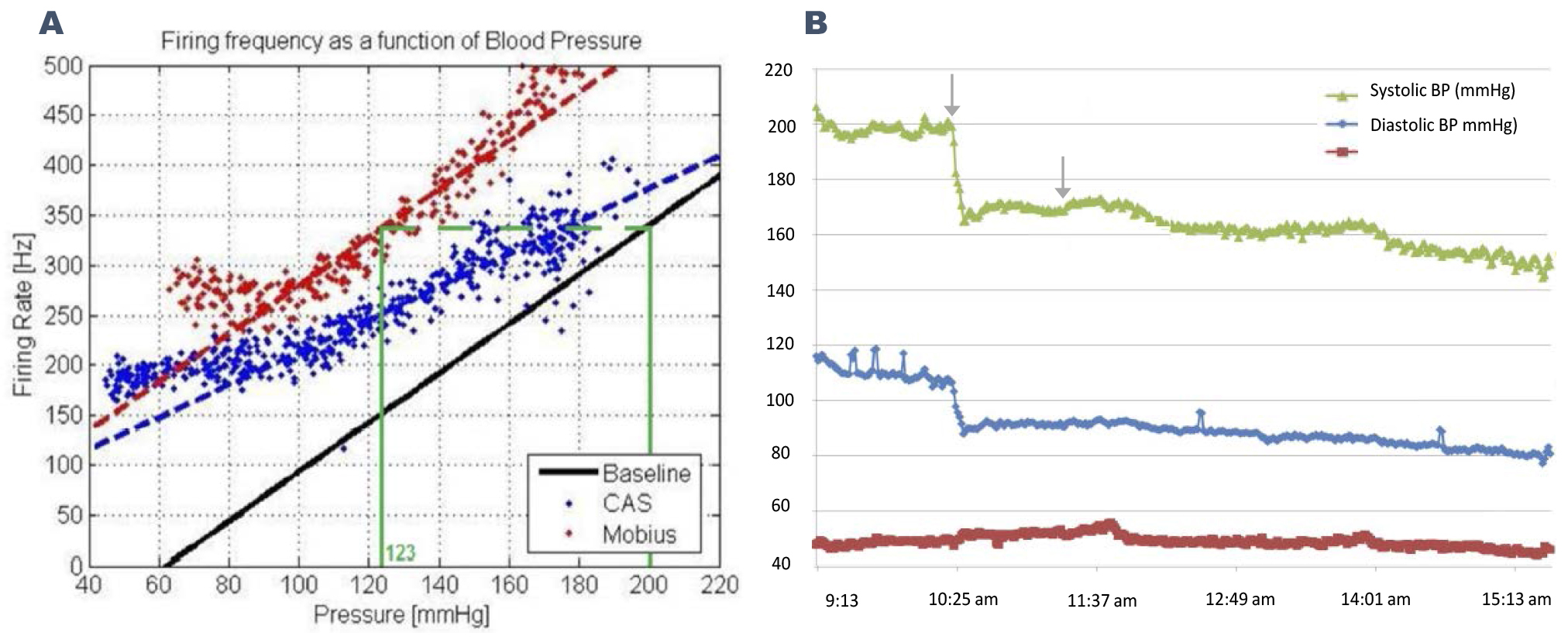

Supplement: Supplementary file 1 — Reproduced from Spiering W, Williams B, van der Heyden J. Endovascular baroreflex amplification for resistant hypertension: a safety and proof-of-principle clinical study. Lancet. 2017;390(10113):2655–61(Fig. S1-S2). DOI: https://doi.org/10.1016/S0140-6736(17)32337-1. Effect of MobiusHD implantation in canine model. (A) Firing rate of the canine baroreceptor after implantation of the MobiusHD device (red) compared to a conventional carotid artery stent (blue) at different blood pressure levels. Maximal nerve activity as well as the baroreceptor response to increases in blood pressure is higher after MobiusHD implantation. (B) After deployment of the first MobiusHD device (first arrow), systolic and diastolic BP drop immediately. Deployment of a second MobiusHD device in the contralateral carotid sinus (second arrow) does not lower BP any further. Heart rate remains unchanged. BP = blood pressure. CAS = carotid artery stent. (JPEG 619 kb) [file 11906_2018_840_MOESM1_ESM.jpg]

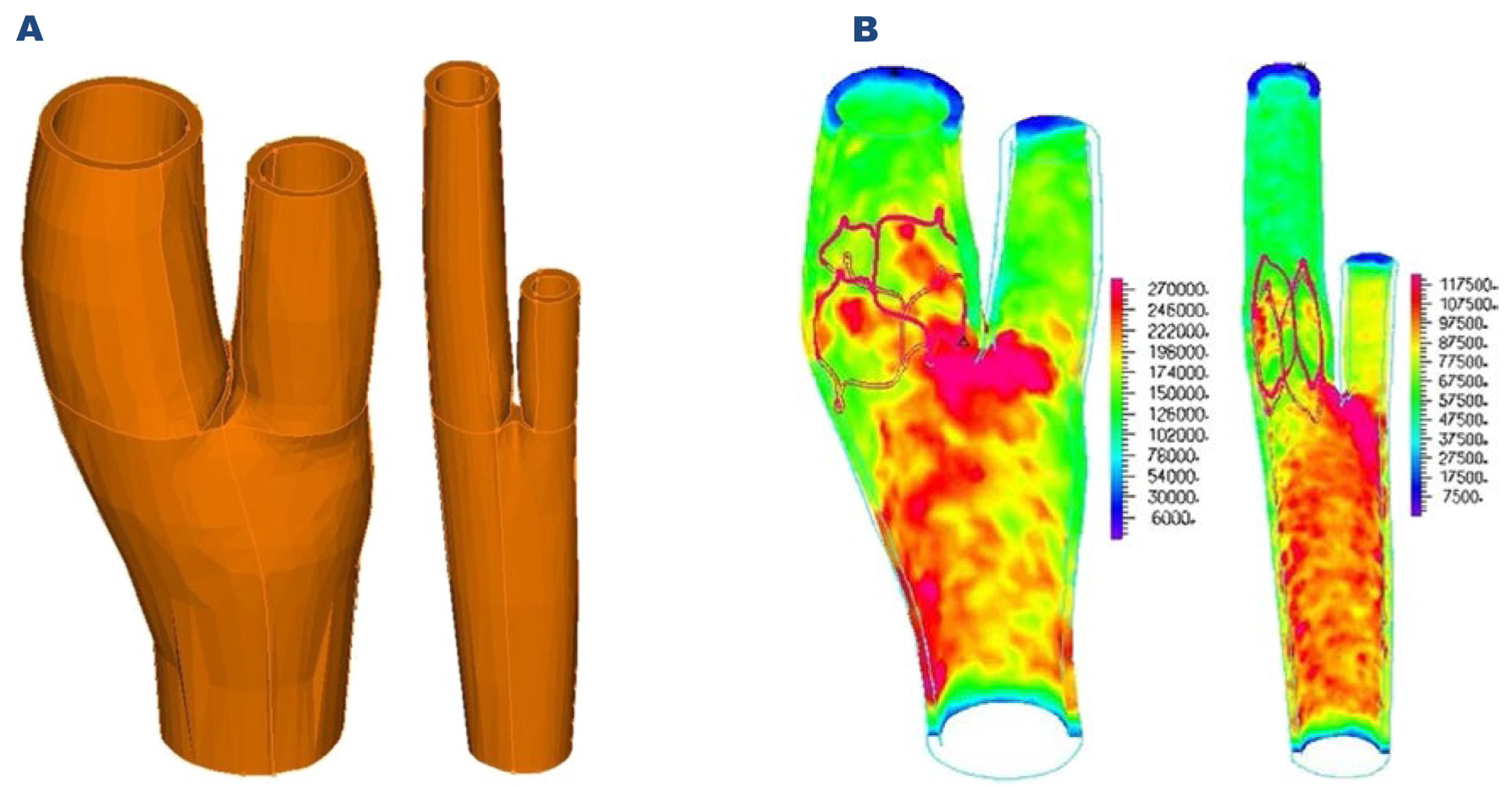

Supplement: Supplementary file 2 — Reproduced from Peter DA, Alemu Y, Xenos M. Fluid structure interaction with contact surface methodology for evaluation of endovascular carotid implants for drug-resistant hypertension treatment. Journal of Biomedical Engineering. 2012:134;041001–2/5. DOI: 0.1115/1.4006339. (A) The two models of the carotid bifurcation used to perform simulations: the left representing an average carotid artery, the right representing the clinical worst-case scenario devoid of a typical sinus, smaller in dimensions and internal and external carotid arteries aligned almost in parallel position. (B) Wall stress distribution (mapped in units of Pa) with the MobiusHD implanted in the carotid sinus of the two models, showing the regions of high wall stress. (JPEG 612 kb) [file 11906_2018_840_MOESM2_ESM.jpg]
